# Effects of low-level laser therapy versus soft occlusive splints on mouth opening and surface electromyography in females with temporomandibular dysfunction: A randomized-controlled study

**DOI:** 10.1371/journal.pone.0258063

**Published:** 2021-10-01

**Authors:** Tamer Shousha, Mohamed Alayat, Ibrahim Moustafa

**Affiliations:** 1 Department of Physiotherapy, College of Health Sciences, University of Sharjah, Sharjah, United Arab Emirates; 2 Faculty of Physical Therapy, Cairo University, Cairo, Egypt; 3 Physical Therapy Department, Faculty of Applied Medical Sciences, Umm Al-Qura University, Mecca, KSA; Prince Sattam Bin Abdulaziz University, College of Applied Medical Sciences, SAUDI ARABIA

## Abstract

**Background:**

Low level lasers have been used as an alternative pain relief therapy for muscle and joint pain, since it induces analgesic, anti-inflammatory, and biomodulation effects of the physiological cell functions. The effectiveness of low-level laser therapy in temporomandibular joint dysfunction (TMD) treatment, however, is not well established. Although Surface electromyography (sEMG) has been suggested as a complementary means in TMD diagnosis, the effect of conservative treatments on muscle activity has not yet been thoroughly correlated with (sEMG) findings.

**Purpose:**

To assess the efficacy of low-level laser therapy (LLLT) as compared to occlusive splint therapy (OST) on the TMJ opening index (TOI) and sEMG of masticatory muscles.

**Materials and methods:**

112 female subjects suffering from unilateral myogenous TMD, aged 21–30 years-old, were recruited and divided into three groups: LLLT; soft occlusive splint therapy OST and a waitlist group as controls.

**Outcome measures:**

TMJ opening index (TOI), Visual analogue scale (VAS), surface electromyography (sEMG).

**Results:**

A significant reduction was reported in TOI, VAS and the sEMG within the LLLT and OST groups as well as significant decrease in all outcomes between groups in favor of the LLLT group (P< 0.0001). Meanwhile, there was a weak significant difference within the control group probably attributed to the analgesic. Post-hoc pairwise comparisons between groups [control vs occlusive splints, control vs low-level laser and low-level laser vs occlusive splints] revealed significant differences in the VAS and TOI [P = 0.0001; 95% CI: 0.9–2.2, 1.61–4.01, 0.65–1.96].

**Conclusions:**

Findings support an evident short term therapeutic effect of the LLLT on improving VAS, TOI and sEMG in females suffering from myogenous TMD.

## Introduction

Temporomandibular dysfunction (TMD) describes clinical conditions involving the temporomandibular joint (TMJ), muscles of mastication, as well as associated tissues [1].

The prevalence of TMD has been reported as 8% to 15% with females affected more than males [2, 3] and peak prevalence of around 35 to 45 years [4].

TMD has been considered a major health concern and a main cause of chronic orofacial pain having negative impact on daily living activities and life quality, Whereas more than a quarter of the general population are subject to TMD at some point in their lives [5].

There is lack of data regarding the prevalence of TMD in the UAE but recently a study reported 41% of the study sample to experience varying degrees of TMJ disorders [6].

Manifestations of TMD include pain around the TMJ, limitations in jaw motion, and TMJ sounds such as clicking or crepitus with movement [1].

In addition, TMD was strongly correlated with neck disability, correlating changes accompanying jaw dysfunction to changes attributed to from neck disability [7].

Regarding treatment, although surgery was once more prevalent, it is now often considered after evidence-based conservative treatment has failed. Non-surgical treatment includes education, behavior modification, drug treatment, occlusal splint, and physiotherapy [8–10].

Currently, physiotherapy (PT) is used to manage TMD aiming to restore normal mandibular function through pain relief, decreasing inflammation and promoting tissue healing.

PT interventions, include manual therapy, exercises [10], ultrasound as well as laser therapy [8, 10, 11].

Low level laser therapy (LLLT) has been recommended for treatment of TMD symptoms [12–14].

The main physiological effects of (LLLT) include bio simulation, promoting tissue regeneration, analgesia, and anti-inflammatory action [14].

Even though LLLT is a commonly used physiotherapy treatment, only few studies discussed its use in the management of TMD [15–21]. LLLT is a non-thermal modality reduce inflammation through inhibition of PEG2 formation and suppression of cyclooxygenase 2 [22, 23]. However, the mechanism underlying the therapeutic effects of LLLT is still under debate [18]. Many prospective randomized controlled clinical trials (RCTs) and retrospective clinical trials have been performed to evaluate this procedure. However, no consensus has been achieved to date [24].

In addition, there is still limited evidence to support clinical effectiveness due to the lack of agreement in defining TMD, inclusion and exclusion criteria and use of valid and reliable outcome measures [25].

Also, despite improving, still many practicing dentists, physicians, and physical therapists (PTs) are not formally trained in the diagnosis and treatment of TMD [26, 27]. In addition, there is a need to objectively guide both assessment and treatment procedures.

Surface electromyography (sEMG) has been proposed as a noninvasive tool in TMD objective assessment providing muscular activity at rest and during functional activities [26, 28, 29].

Unfortunately, the effect of LLLT application on sEMG activity of masticatory muscles has been poorly investigated [29, 30].

### Purpose of the study

To assess the efficacy of low-level laser therapy (LLLT) as compared to occlusive splint therapy (OST) on the TMJ opening index (TOI) and sEMG of masticatory muscles.

## Materials and methods

This study was conducted in accordance with the Declaration of Helsinki and was approved by the research ethics committee, Faculty of Physical Therapy, Cairo University, Egypt; No.: P.T.REC/012/002876 and registered in the ClinicalTrials.gov: NCT04831346.

We were unfortunate to have the registration delayed as the research team member responsible for the registration suffered from the COVID infection and the team discovered the issue later.

The authors confirm that all ongoing and related trials for this intervention are registered.

### Design

A double-blinded parallel group randomized controlled trial. An external assessor with more than 10 years clinical experience, blinded to both treatment and results was recruited to deliver the LLLT.

Before the study started, we clearly explained the procedures with the subjects and 112 females provided their signed consent to enroll.

### Subjects

One hundred and twelve females with unilateral myogenous TMD, aged 21–30 years-old, were recruited and divided into three group: 37 patients were assigned to the LLLT; 37 patients received a soft occlusive splint therapy OST; 38 were placed on the waitlist as controls.

Subjects were instructed by the dentist of using paracetamol which is a very mild analgesic only in cases of urgent need.

The study was conducted in the outpatient clinic of the Faculty of Physical Therapy, Cairo University from November 2020 to February 28, 2021. Based on the current COVID -19 pandemic, subjects were treated individually in a special ward maintain the social distancing regulations and sanitization protocols.

All subjects included were referred from the faculty of dentistry dental hospital after thorough examination to ensure meeting the inclusion criteria according to Monaco et. al, 2012 [31].

Inclusion was limited to females less than 30 years of age, diagnosed with unilateral myogenous TMD, having complete permanent dentition and showing normal occlusion.

Subjects were excluded if they met one or more of the following criteria:

Prior experience of Laser therapy, systemic diseases (rheumatoid arthritis, ankylosing spondylitis, diabetes… etc.; history of trauma in the TMJ or cervical regions; neurological disorders, muscular diseases; cervical pain; bruxism, pregnancy; currently on medication: analgesic, anti-inflammatory, muscle relaxants or anti-depressants) current use of dental prosthetics; previous orthodontal treatments; or fixed restorations affecting occlusal surfaces.

### Randomization

**Fig 1:**
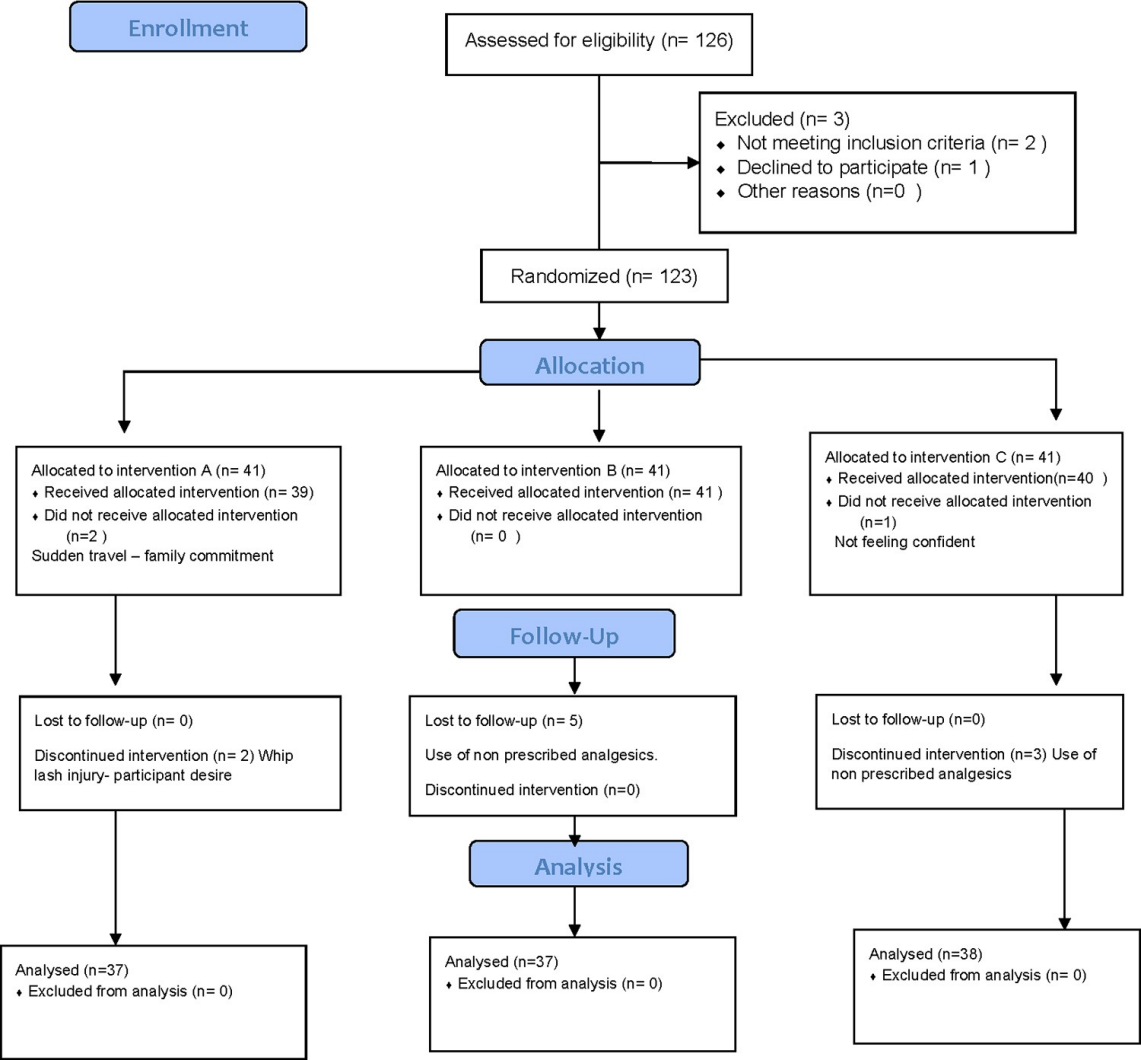
Randomization flow chart.

One hundred and twenty-six females with TMD were considered for this study whereas, two not meeting our selection criteria and one declined to enroll. The remaining one hundred and twenty-three subjects were assigned randomly into three groups of equal numbers. During allocation three did not receive allocated intervention (two due to sudden travel and family commitment as well as one for not feeling confident about treatment). Later during follow up ten subjects were discontinued (two for whip lash injury and participant desire and five for using analgesics different from the dentist prescription). Finally, one hundred twenty-one subjects were considered for analysis. (Fig 1: Randomization flow chart).

Randomization and allocation were done by an independent therapist in the clinic blinded from the purpose and outcomes of the study.

Randomization was limited to of permuted blocks of different sizes to ensure an equal number of allocations in each group. Each random block was stored in a locked drawer until required. They were stored as opaque sealed envelopes consecutively sequenced and numbered. The researcher opens the subsequent envelope after each participant formally enters the trial. Participants and the therapist were blinded to the study hypotheses.

### Outcome measures

#### Primary outcome

The Temporomandibular joint opening index (TOI):

Limitation of mandible movement is one of the signs of TMDs, evaluated by measurement of maximum voluntary mouth opening. However, the influence of gender, age [32], ramus length [33] and an inability to discriminate between diagnostic groups, limits its usefulness [34].

A new temporomandibular index was designed and was reported not to be influenced by the above variables [35] having a greater diagnostic value than linear mouth opening [34].

The temporomandibular opening index (TOI) is calculated by the formula [35]:

$$TOI=\frac{Passive\;opening_{mm}‐Maximum\;voluntary\;opening_{mm}}{Passive\;opening_{mm}+Maximum\;voluntary\;opening_{mm}}X\;100$$

Linear measurement of mouth opening is one of the methods of assessing limitation of mandibular movement, considered one of the cardinal signs of TMD [36] however, the influence of gender, age [32], ramus length [33], and an inability to discriminate between diagnostic groups, limits its usefulness [37].

Active and passive mouth opening was assessed by a Boley gauge (Electronic Digital Caliper, CE Company, Japan).

### Secondary outcomes

1- Surface EMG (sEMG):

Surface electromyography (sEMG) provides non-invasively information on muscle properties. The EMG allows the evaluation of muscle function and dysfunction at rest as well as during occlusion which aids in the differential diagnosis and monitoring of TMD during therapy [38].

For recording, surface electromyography (Myotronics-Noromed, Inc., Tukwila WA, USA), with 8-channels, simultaneous acquisition, common grounding to all channels, and filters of 50 Hz electromyography with disposable electrodes was used.

Subjects were seated on chairs with back and head rests to allow assessment from a relaxed position.

The right masseter (RM), left masseter (LM), right anterior temporal (RAT), left anterior temporal (LAT), right sternocleidomastoid (RSM), and left sternocleidomastoid (LSM) muscles were recorded. The sEMG recordings and muscle activity was expressed as the root mean square (rms) of the amplitude, expressed in μV.

Electrode positions were adopted from Castroflorio et al., 2005 for the left and right masseter muscles (LM, RM) and the left and right anterior temporal muscles (LAT, RAT) [28] as well as Falla et al., 2002 for the left and right sternocleidomastoid muscle (LSM, RSM) [39].

The ground electrode was positioned on the forehead as a common reference to the amplifier’s differential input.

**2- Visual analogue scale**

Pain intensity was assessed by the Visual Analogue Scale (VAS) where no pain was rated as 0 mm and worst possible pain as 100 mm [40, 41].

The pain VAS is a reliable measure of pain intensity [40] with a 10 centimeters (100 mm) continuous scale attached by 2 verbal descriptors, one for each symptom extreme [40, 41].

For pain intensity, the scale is most anchored by “no pain” (score of 0) and “pain as bad as it could be” or “worst imaginable pain” (score of 100) [42].

The VAS only requires the ability to use a ruler to measure the distance to determine a score.

### Intervention

1- LLLT:

A low-level gallium arsenide diode (Biolase, USA) at a 940 nm wavelength with 0.2 W output power and 2 J energy. The device was calibrated, and the probe was disinfected prior to every treatment.

The Masseter and Temporalis muscles were bilaterally assessed with constant pressure to define tenderness.

LLLT was applied perpendicular to each tender point of the intended muscles for 10 seconds with an energy density of 2.5 J/cm^2^.

Sessions were scheduled 3 days a week (every other day) for a total of 10 sessions [43].

2- Occlusive splint:

A soft occlusal splint (vacuum-formed) made from a 2-mm-thick elastic rubber sheets was used [44].

Splints were individually designed for the upper arch of each patient. An alginate imprint of the maxillary arch was taken to fabricate a master cast of the maxilla.

A vacuum pressure device was utilized for molding the rubber sheets (13 x 13 cm /2-mm thickness).

Sheets were removed after it has been appropriately adjusted to the mold in the vacuum former. Edges were properly trimmed, and the palate part was detached to obtain the end shape.

Participants were instructed to wear the splint at all times except during mealtimes and oral hygiene.

### Sample size

To calculate the sample needed, estimates for the means and standard deviations were obtained from a pilot study of 15 patients that received this treatment protocol in the period from November 2020 to February 28, 2021. The mean difference and standard deviation of the TMJ opening were estimated to be 1.91 and 0.41, respectively.

This was in agreement with Alacreu et al., 2014 [45] identifying the minimal detectable change (MDC) as 1.88.

The G* power software was utilized to detect a total sample size of 81 participants. Accordingly, considering a 5% level of significance and statistical power of 80% and an effect size of 0.4, at least 27 subjects per group were required for the current study. An excess of 20% was considered accounting for possible dropouts. The software is available online from the Universität Düsseldorf web site (https://www.psychologie.hhu.de/arbeitsgruppen/allgemeine-psychologie-und-arbeitspsychologie/gpower).

### Statistical analysis

Before the final analysis, the Shapiro-Wilk test revealed the data to be normally distributed (P>0.05). Similarly, the Levene’s test assessed homogeneity of variance revealing no significant difference (P>0.05). Accordingly, parametric analysis was done with the SPSS Package version 25 for Windows (SPSS, Inc., Armonk, NY: IBM Corp). Data was expressed as mean and standard deviation.

A 3 x 2 MANOVA mixed design was used for comparing variables in different groups and measurement times.

Bonferroni correction was used for post-treatment pairwise group comparisons for variables whose F value from MANOVA test was significant. The significance was reported as (P ≤ 0.05).

## Results

One hundred and twelve subjects were included in this study during the period from November 2020 to June 2021. Participants were randomly distributed to three groups. The one-way ANOVA revealed no significant differences in demographic data (Table 1).

**Table 1:** Comparing demographic data (mean values).

Variable	Group (Mean ±SD)			P-value
	LLLT	OST	Control	
	(n = 37)	(n = 37)	(n = 38)	
Age [Year]	26.21 ±0.64	25.73 ±0.35	27.33 ±0.42	0.071
Weight [kg]	57.24 ±2.73	55.33 ±2.77	56.93 ±1.97	0.832
Height [cm]	161.52 ±6.84	159.32 ±6.06	160.92 ±7.12	0.662
BMI [kg/m^2^]	21.64 ±1.70	22.13 ±2.16	21.50 ±2.24	0.554

SD: standard deviation, P-value: probability value, BMI: Body Mass Index, *Significant (P<0.05)

Regarding within and between group analyses, a 3 x 2 MANOVA mixed design was used for comparing variables in different groups and measurement times (Table 2).

**Table 2:** Within and between group comparisons (Mixed design MANOVA).

Variable		Group (Mean ±SD)			P-value
		LLLT	OST	Control	
		(n = 37)	(n = 37)	(n = 38)	
VAS	Baseline	8.41 ±0.31	7.35 ±0.78	6.51±1.36	0.415
	Post-treatment	2.11 ±0.41	3.22 ±0.19	5.14 ±0.26	0.0001*
	Mean difference	6.3	4.13	1.37	
	Improvement %	72.65%	56.19%	21.04%	
	P-value	0.0001*	0.0001*	0.042*	
TOI	Baseline	16.3 ±1.17	17.2 ±0.16	16.2 ±0.98	0.510
	Post-treatment	3.6 ±0.27	6 ±0.13	13.1±0.18	0.0001*
	Mean difference	12.7	11.2	0.82	
	Improvement %	77.9%	65.11%	19.1%	
	P-value	0.0001*	0.0001*	0.0461*	
sEMG					
RAT	Baseline	2.76 ±1.67	2.68 ±1.23	2.75 ±1.63	0.790
	Post-treatment	1.59 ±1.03	1.71 ±1.10	2.34 ±1.71	0.027*
	Mean difference	1.17	0.97	0.41	
	Improvement %	42.39%	36.19%	18.99%	
	P-value	0.023	0.003*	0.044*	
LAT	Baseline	2.86 ±1.64	2.79 ±1.71	2.78 ±1.80	0.831
	Post-treatment	1.67 ±1.06	1.81 ±1.13	2.21 ±1.13	0.0001*
	Mean difference	1.19	0.98	0.57	
	Improvement %	41.60%	35.12%	19.85%	
	P-value	0.001*	0.001*	0.047*	
RM	Baseline	1.49±0.98	1.45±1.09	1.47±1.11	0.798
	Post-treatment	1.18±0.72	1.34±0.99	1.24±1.01	0.0001*
	Mean difference	0.31	0.11	0.23	
	Improvement %	69.80%	34%	15.64%	
	P-value	0.0001*	0.001*	0.062	
LM	Baseline	1.57±0.89	1.54±0.92	1.56±0.86	0.0001*
	Post-treatment	1.15±0.75	1.20±0.76	1.39±0.71	
	Mean difference	0.42	0.34	0.17	
	Improvement %	36.52	22.07	10.89	
	P-value	0.001*	0.001*	0.066	
RSM	Baseline	2.39±1.76	2.41±1.45	2.28±1.98	0.0001*
	Post-treatment	1.47±1.14	1.91±1.21	2.01±1.67	
	Mean difference	0.92	0.50	0.27	
	Improvement %	38.49%	20.74%	11.84%	
	P-value	0.001*	0.001*	0.721	
LSM	Baseline	2.43±1.57	2.39±1.62	2.41±1.81	0.0001*
	Post-treatment	1.29±1.12	1.78±1.24	2.12±1.4	
	Mean difference	1.14	0.61	0.29	
	Improvement %	46.91%	25.52%	12.03	
	P-value	0.001*	0.001*	0.742	

SD: standard deviation, P-value: probability value

*Significant [P<0.05], VAS: visual analogue scale, TOI: TMJ opening Index, SEMG: Surface EMG, RAT: Right Anterior Temporalis, LAT: Left Anterior Temporalis, RM: Right Masseter, LM: Left Masseter, RSM: Right Sternomastoid, LSM: Left Sternomastoid

The Multivariate tests demonstrated statistically significant (P<0.05) differences due to main effects of tested groups (F16.486; P = 0.0001; Partial η2 = 0.291), measuring time (F = 224.795; P = 0.0001; Partial η2 = 0.842), and group x time interaction (F = 341.00; P = 0.0001; Partial η2 = 0.242).

Post-treatment, within group analysis (Table 2) demonstrated a significant decrease (P<0.05) in VAS, TOI and sEMG within the LLLT and OST groups as well as a non-significant decrease in VAS, TOI, sEMG values within the Control group.

Between groups analysis (Table 2) demonstrated a non-significant difference (P>0.05) in pre-treatment mean values. However, there were significant differences (P<0.05) in the post-treatment mean values of VAS, TOI, sEMG.

Post-hoc pairwise analysis (Table 3), revealed significant differences (P<0.05).

**Table 3:** Post-hoc pairwise comparisons between groups.

Variables		Bonferroni correction		
		Control vs. OST	Control vs. LLLT	LLLT vs. OST
VAS	Mean difference	1.92	3.03	1.11
	95% CI	0.9–2.2	1.61–4.01	0.65–1.96
	P-value	0.0001*	0.0001*	0.0001*
TOI	Mean difference	7.1	9.5	2.4
	95% CI	0.9–2.2	1.61–4.01	0.65–1.96
	P-value	0.0001*	0.0001*	0.0001*
RAT	Mean difference	0.63	0.75	0.12
	95% CI	0.27–1.36	0.21–1.4	0.05–0.15
	P-value	0.001*	0.001*	0.001*
LAT	Mean difference	0.4	0.54	0.14
	95% CI	0.01–0.91	0.25–0.97	0.01–0.09
	P-value	0.0001*	0.0001*	0.0001*
RM	Mean difference	0.1	0.06	0.16
	95% CI	0.05–0.94	0.01–0.12	0.05–0.96
	P-value	0.0001*	0.001*	0.0001*
LM	Mean difference	0.19	0.24	0.05
	95% CI	0.05–0.21	0.1–0.21	0.02–0.1
	P-value	0.002*	0.001*	0.001*
RSM	Mean difference	0.1	0.54	0.44
	95% CI	0.05–0.94	0.21–0.92	0.21–0.91
	P-value	0.0001*	0.0001*	0.0001*
LSM	Mean difference	0.34	0.83	0.49
	95% CI	0.1–0.71	0.31–1.2	0.21–1.2
	P-value	0.0001*	0.002*	0.002*

CI: Confidence interval

*Significant [P<0.05], VAS: visual analogue scale, TOI: TMJ opening Index, SEMG: Surface EMG, RAT: Right Anterior Temporalis, LAT: Left Anterior Temporalis, RM: Right Masseter, LM: Left Masseter, RSM: Right Sternomastoid, LSM: Left Sternomastoid.

## Discussion

This study was the first phase for evaluating the effect of the LLLT compared to soft occlusive splints during a one year follow up. The purpose of the current phase was to compare the short-term effect of LLLT to soft occlusive splints on muscular activity in cases of TMD.

The effect of conservative measures in treatment of TMD is controversial across literature, whereas moderate to very low-quality evidence regarding the effectiveness of occlusal splint therapy in the treatment of TMD was reported [46, 47], equivalent with other therapeutic modalities [43, 48, 49] or with no effect reported [50].

On the other hand, although previous studies reported positive effects of physiotherapeutic modalities on pain and function in cases of TMD [10, 13, 31, 51] still, there is few to no evidence supporting the use of electro physical modalities [52].

With regards to the efficacy of LLLT, although McNeely et al. (2006) found no evidence to support the use of any electrophysical modalities commonly used by physical therapists [36], where laser intensity is reduced by 90% at a tissue depth of 1 cm [53] the results of the current study came in accordance with previous studies revealing the efficacy of LLLT in reducing pain [12, 49, 51] and sEMG of masticatory muscles [30, 54].

This can be simply attributed to the bio-stimulating and analgesic effects of LLLT through direct irradiation without thermal response [54] and to the application to the Masseter and Temporalis muscles which is rather superficial in comparison to other deep structures as the lateral Pterygoid [53].

The physiological effects of LLLT are characterized by increased production of â-endorphins and control of the production of prostaglandins [55, 56]. The application therefore reduces pain while simultaneously reducing muscle contraction.

In addition, since TMD is considered to cause consequent imbalance of the whole body [57], it presents with pain in the cervical spine [58] where the myo-functional disturbances of the TMJ and craniovertebral joints may lead to overlapping complaints [59].

On the other hand, although our results revealed reduction in sEMG of the Sternomastoid muscles, this was not in agreement with Monaco et al., 2012 reporting a non-significant reduction in sEMG of the Sternomastoid muscles [31]. This can be attributed to the difference in modality utilized since their study considered the effect of TENS. Another cause might be related to the inclusion criteria as their sample criteria did not have pain rather than remission within the last three months.

With regards to occlusive splinting, our findings came in agreement with Seifeldin et al., 2015 reporting soft and hard splinting to cause improvements in visual analog scores, tenderness of masticatory muscles and range of mouth opening [44].

But we were not in agreement with Al-Moraissi et al., 2020 reporting hard stabilization splints to have better results in cases of myogenous TMD [46]. We believe this might be due to the different timing of application since our study advocated wearing of the splint throughout the day except during mealtimes and oral hygiene.

In addition, despite our findings reveal a statistically significant difference in favor of the LLLT group as compared to the OST and control, it must be noted that this effect might be evident only in the short term owing to the substantial association between the effectiveness of stabilization splints, and the overall duration of follow-up [46].

Another interesting fact to note is the less significant improvement of the control group which can be attributed to the low-grade analgesic prescribed by the dentist.

### Clinical implications

The findings of this study provide objective evidence of the effect of using low-level laser in relieving pain and improving mouth opening as guided by the sEMG findings.

### Limitations

This study included only female participants therefore the effect of gender difference was not considered. Another limitation is the inability to add a sham group based on the lack of psychological assessment tools required to assess the psychological influence on therapy.

### Recommendations

Further studies should focus on including participants of both sexes, different age groups and BMI levels. Further studies are also required to evaluate the effect of applying LLLT on the long term with a follow-up period.

## Conclusion

The findings of our study showed the evident short term therapeutic effect of the low-level laser therapy on improving visual analogue scale, temporomandibular opening index and surface electromyography in females with myogenous TMD.

## Supporting information

S1 Checklist CONSORT 2010 checklist of information to include when reporting a randomised trial*. (DOC)

S1 Study protocol (DOCX)
