# Supplementary material for: Effects of low-level laser therapy versus soft occlusive splints on mouth opening and surface electromyography in females with temporomandibular dysfunction: A randomized-controlled study
Source: PLoS One. 2021 Oct 1;16(10):e0258063. doi: 10.1371/journal.pone.0258063 (PMC8486092; doi:10.1371/journal.pone.0258063)
Supplement: S1 Study protocol — (DOCX) [file pone.0258063.s002.docx]

**Protocol Title:**

**Effects of Low-Level Laser Therapy Versus Soft Occlusive Splints on Mouth Opening and Surface Electromyography in Temporomandibular Dysfunction: A Randomized-controlled Study**

**Submitted by:**

**Dr. Tamer M. Shousha^1,2^**

**Dr. Mohamed S. Alayat^2,3^**

**Dr. Ibrahim M. Moustafa^1,2^**

^1^Department of Physiotherapy, College of Health Sciences, University of Sharjah, United Arab Emirates

^2^Faculty of Physical Therapy, Cairo University, Cairo, Egypt

^3^Physical Therapy Department, Faculty of Applied Medical Sciences, Umm Al-Qura University, KSA

**Effects of Low-Level Laser Therapy Versus Soft Occlusive Splints on Mouth Opening and Surface Electromyography in Temporomandibular Dysfunction: A Randomized-controlled Study**

**Introduction**

Temporomandibular dysfunction (TMD) describes clinical conditions involving the temporomandibular joint (TMJ), muscles of mastication, as well as associated tissues.^1^

The prevalence of TMD has been reported of 8% to 15% with females affected more than males^2^ with the peak prevalence around 35 to 45 years.^3^

TMD has been considered a major health concern and a main cause of chronic orofacial pain having negative impact on daily living activities and life quality, Whereas more than a quarter of the general population are subject to TMD at some point in their lives.^4^

Manifestations of TMD include pain around the TMJ, limitations in jaw motion, and TMJ sounds such as clicking or crepitus with movement.^1^

Physiotherapy (PT) is currently considered as a managing strategy for TMD disorders aiming to restore normal mandibular function through pain relief, decreasing inflammation and promoting tissue healing.

PT interventions, include manual therapy, exercises, and ultrasound as well as laser therapy. ^5–7^

One of the recommended PT methods for pain relief, decreasing inflammation and promoting tissue healing is Low level laser therapy [LLLT].^8–10^ however, only few studies discussed its use in the management of TMD. ^11–16^

**Statement of the problem**

There is still limited evidence to support clinical effectiveness in TMD treatment due to the lack of agreement in defining TMD, inclusion and exclusion criteria and use of valid and reliable outcome measures.^17^

Also, despite improving, still many practicing dentists, physicians, and physical therapists (PTs) are not formally trained in the diagnosis and treatment of TMD.^18,19^

There is a need to objectively guide both assessment and treatment procedures.

One of the currently used methods is Surface electromyography (sEMG) that has been proposed as a noninvasive tool in TMD objective assessment providing muscular activity at rest and during functional activities. ^18,20,21^

Unfortunately, the effect of LLLT application on sEMG activity of masticatory muscles has been poorly investigated.^21,22^

**Purpose of the study**

To assess the efficacy of low-level laser therapy (LLLT) as compared to occlusive splint therapy (OST) on the TMJ opening index (TOI) and sEMG of masticatory muscles.

**Materials and methods**

**Design**

A double-blinded parallel group randomized controlled trial. An external assessor with more than 10 years clinical experience, blinded to both treatment and results was recruited to deliver the LLLT.

**Subjects**

This study will be conducted in accordance with the Declaration of Helsinki after approval is obtained from the research ethics committee, Faculty of Physical Therapy, Cairo University, Egypt. The protocol will be registered in the ClinicalTrials.gov.

Sample size was calculated through estimates for the means and standard deviations that will be obtained from a pilot study on 15 patients to receive the treatment protocol.

**Inclusion will be limited to**:

Females less than 30 years of age, diagnosed with unilateral myogenous TMD, having complete permanent dentition and showing normal occlusion.

Subjects were excluded if they met one or more of the following criteria:

Prior experience of Laser therapy, systemic diseases (rheumatoid arthritis, ankylosing spondylitis, diabetes… etc.; history of trauma in the TMJ or cervical regions; neurological disorders, muscular diseases; cervical pain; bruxism, pregnancy; currently on medication (analgesic, anti-inflammatory, muscle relaxants or anti-depressants) current use of dental prosthetics; previous orthodontal treatments; or fixed restorations affecting occlusal surfaces.

**Outcome measures:**

**Primary outcome:**

The temporomandibular opening index (TOI) is calculated by the formula ^23^ :

TOI= Passive opening _mm_ -Maximum voluntary opening _mm_ X 100

Passive opening _mm_ + Maximum voluntary opening _mm_

**Secondary outcomes:**

1. Surface EMG (sEMG):

For recording, a surface electromyograph (Myotronics-Noromed, Inc., Tukwila WA, USA), with 8-channels, simultaneous acquisition, common grounding to all channels, and filters of 50 Hz electromyography with disposable electrodes was used.

Subjects will be seated on chairs with back and head rests to allow assessment from a relaxed position.

The right masseter (RM), left masseter (LM), right anterior temporal (RAT), left anterior temporal (LAT), right sternocleidomastoid (RSM), and left sternocleidomastoid (LSM) muscles will be recorded.

Electrode positions are adopted from Castroflorio et al., 2005 for the left and right masseter muscles (LM, RM) and the left and right anterior temporal muscles (LAT, RAT) ^20^ as well as Falla et al., 2002 for the left and right sternocleidomastoid muscle (LSM, RSM). ^24^

The ground electrode will be positioned on the forehead as a common reference to the amplifier’s differential input.

1. Visual analogue scale

Pain intensity will be assessed by the Visual Analogue Scale (VAS) where no pain is rated as 0 mm and worst possible pain as 100 mm.^25,26^

**Procedures**:

Laser group: A low-level gallium arsenide diode (Biolase, USA) at a 940 nm wavelength with 0.2 W output power and 2 J energy will be used. The device will be calibrated, and the probe to be disinfected prior to every treatment.

The Masseter and Temporalis muscles will be bilaterally assessed with constant pressure to define tenderness.

LLLT will be applied perpendicular to each tender point of the intended muscles for 10 seconds with an energy density of 2.5 J/cm^2^.

Sessions will be scheduled 3 days a week (every other day) over a period of 4 months.

1. Occlusive splint:

A soft occlusal splint (vacuum-formed) made from a 2-mm-thick elastic rubber sheets was used. ^27^

Splints will be individually designed for the upper arch of each patient. An alginate imprint of the maxillary arch was taken to fabricate a master cast of the maxilla.

Participants will be instructed to wear the splint at all times except during mealtimes and oral hygiene.

**Statistical analysis:**

The SPSS Package version 25 for Windows (SPSS, Inc., Armonk, NY: IBM Corp) will be considered for the analysis.

Data will be tested for homogeneity and a 3 x 2 MANOVA mixed design was used for comparing variables in different groups and measurement times.

**References**

1. De Leeuw R, Klasser GD. *Orofacial Pain: Guidelines for Assessment, Diagnosis, and Management*. Quintessence Chicago; 2008.

2. Dworkin SF, Huggins KH, LeResche L, et al. Epidemiology of signs and symptoms in temporomandibular disorders: clinical signs in cases and controls. *J Am Dent Assoc*. 1990;120(3):273-281.

3. Kraus SL. Characteristics of 511 patients with temporomandibular disorders referred for physical therapy. *Oral Surg Oral Med Oral Pathol Oral Radiol*. 2014;118(4):432-439.

4. Rashid A, Matthews NS, Cowgill H. Physiotherapy in the management of disorders of the temporomandibular joint—perceived effectiveness and access to services: a national United Kingdom survey. *Br J Oral Maxillofac Surg*. 2013;51(1):52-57.

5. Shousha TM, Soliman ES, Behiry MA. The effect of a short term conservative physiotherapy versus occlusive splinting on pain and range of motion in cases of myogenic temporomandibular joint dysfunction: a randomized controlled trial. *J Phys Ther Sci*. 2018;30(9):1156-1160.

6. Glass GE, Glares GA, McGlynn FD. Myofascial pain dysfunction: treatments used by ADA members. *Cranio®*. 1993;11(1):25-29.

7. Lyons MF. Current practice in the management of temporomandibular disorders. *Dent Update*. 2008;35(5):314-318.

8. Khairnar S, Kalyani Bhate SKSN, Kshirsagar K, Jagtap B, Kakodkar P. Comparative evaluation of low-level laser therapy and ultrasound heat therapy in reducing temporomandibular joint disorder pain. *J Dent Anesth pain Med*. 2019;19(5):289.

9. Xu G-Z, Jia J, Jin L, Li J-H, Wang Z-Y, Cao D-Y. Low-level laser therapy for temporomandibular disorders: a systematic review with meta-analysis. *Pain Res Manag*. 2018;2018.

10. Shukla D, Muthusekhar MR. Efficacy of low-level laser therapy in temporomandibular disorders: A systematic review. *Natl J Maxillofac Surg*. 2016;7(1):62.

11. Dostalová T, Hlinakova P, Kasparova M, Rehacek A, Vavrickova L, Navrátil L. Effectiveness of physiotherapy and GaAlAs laser in the management of temporomandibular joint disorders. *Photomed Laser Surg*. 2012;30(5):275-280.

12. De Godoy CHL, da Costa Silva PF, De Araujo DS, et al. Evaluation of effect of low-level laser therapy on adolescents with temporomandibular disorder: study protocol for a randomized controlled trial. *Trials*. 2013;14(1):1-6.

13. Catão MHC de V, Oliveira PS de, Costa R de O, Carneiro VSM. Avaliação da eficácia do laser de baixa intensidade no tratamento das disfunções têmporo-mandibular: estudo clínico randomizado. *Rev CEFAC*. 2013;15(6):1601-1608.

14. Madani AS, Ahrari F, Nasiri F, Abtahi M, Tunér J. Low-level laser therapy for management of TMJ osteoarthritis. *Cranio®*. 2014;32(1):38-44.

15. Pereira TS, Flecha OD, Guimarães RC, et al. Efficacy of red and infrared lasers in treatment of temporomandibular disorders—a double-blind, randomized, parallel clinical trial. *Cranio®*. 2014;32(1):51-56.

16. Seifi M, Ebadifar A, Kabiri S, Badiee MR, Abdolazimi Z, Amdjadi P. Comparative effectiveness of Low Level Laser therapy and Transcutaneous Electric Nerve Stimulation on Temporomandibular Joint Disorders. *J lasers Med Sci*. 2017;8.

17. Fricton JR, Ouyang W, Nixdorf DR, Schiffman EL, Velly AM, Look JO. Critical appraisal of methods used in randomized controlled trials of treatments for temporomandibular disorders. *J Orofac Pain*. 2010;24(2):139.

18. Miller VJ, Bookhan V, Brummer D, et al. Surface EMG of jaw elevator muscles: effect of electrode location and inter‐electrode distance. *J Oral Rehabil*. 2001;32(6):1022-1024.

19. McNeill C, Falace D, Attanasio R. Continuing education for TMD and orofacial pain: a philosophical overview. *J Craniomandib Disord facial oral pain*. 1992;6(2):135-136.

20. Castroflorio T, Farina D, Bottin A, Piancino MG, Bracco P, Merletti R. Surface EMG of jaw elevator muscles: Effect of electrode location and inter-electrode distance. *J Oral Rehabil*. 2005;32(6):411-417. doi:10.1111/j.1365-2842.2005.01442.x

21. Leal De Godoy CH, Motta LJ, Garcia EJ, et al. Electromyographic evaluation of a low-level laser protocol for the treatment of temporomandibular disorder: A randomized, controlled, blind trial. *J Phys Ther Sci*. 2017;29(12):2107-2111. doi:10.1589/jpts.29.2107

22. Shinozaki EB, dos Santos MBF, Okazaki LK, Marchini L, Junior AB. Clinical assessment of the efficacy of low-level laser therapy on muscle pain in women with temporomandibular dysfunction, by surface electromyography. *Brazilian J Oral Sci*. Published online 2016:434-438.

23. Miller VJ, Bookhan V, Brummer D, Singh JC. A mouth opening index for patients with temporomandibular disorders. *J Oral Rehabil*. 1999;26(6):534-537.

24. NEKORA AZAK A, EVLIOGLU G, ORDULU M, et al. Repeatability of surface EMG variables in the sternocleidomastoid and anterior scalene muscles. *Istanbul Tip Fak Derg Cilt*. 2012;69(3):463-468. doi:10.4103/1305-7456.184156

25. Ferraz MB, Quaresma MR, Aquino LR, Atra E, Tugwell P, Goldsmith CH. Reliability of pain scales in the assessment of literate and illiterate patients with rheumatoid arthritis. *J Rheumatol*. 1990;17(8):1022-1024.

26. Scott J, Huskisson EC. Vertical or horizontal visual analogue scales. *Ann Rheum Dis*. 1979;38(6):560.

27. Seifeldin SA, Elhayes KA. Soft versus hard occlusal splint therapy in the management of temporomandibular disorders (TMDs). *Saudi Dent J*. 2015;27(4):208-214.
